# Learning Optimal Fin-Ray Finger Design for Soft Grasping

**DOI:** 10.3389/frobt.2020.590076

**Published:** 2021-02-12

**Authors:** Zhifeng Deng, Miao Li

**Affiliations:** ^1^Learning Algorithms and Soft Manipulation Laboratory, The Institute of Technological Science, School of Power and Mechanical Engineering, Wuhan University, Wuhan, China; ^2^Wuhan Cobot Technology, Wuhan, China

**Keywords:** soft hands, robotic grasping, soft finger design, grasp quality, grasp quality criterion

## Abstract

The development of soft hands is an important progress to empower robotic grasping with passive compliance while greatly decreasing the complexity of control. Despite the advances during the past decades, it is still not clear how to design optimal hands or fingers given the task requirements. In this paper, we propose a framework to learn the optimal design parameter for a fin-ray finger in order to achieve stable grasping. First, the pseudo-kinematics of the soft finger is learned in simulation. Second, the task constraints are encoded as a combination of desired grasping force and the empirical grasping quality function in terms of winding number. Finally, the effectiveness of the proposed approach is validated with experiments in simulation and using real-world examples as well.

## 1. Introduction

Soft robotics is one of the most fast-growing area in robotics. This is in part due to the breaking idea of building robots from highly compliant material similar as living organisms (Laschi et al., 2016). More importantly, soft robots allows for increased flexibility and adaptability for accomplishing complex tasks that is impossible for traditional rigid robots. Therefore, soft robots brings up the potential to push the boundaries of current robot abilities (Laschi et al., 2016). Previous work in this domain mostly focuses on the design of new types of soft robots (Lipson, 2014; Rus and Tolley, 2015; Hughes et al., 2016), including novel driving actuators (Polygerinos et al., 2015b; Zhao et al., 2016), bio-inspired structure and mechanism (Cutkosky and Kim, 2009; Lipson, 2014; Manti et al., 2015; Hughes et al., 2016), special design and control methods (Hiller and Lipson, 2011), and stretchable electronics (Rogers et al., 2010). Despite these achievements, the relation between given task at hand and the correct robot embodiment for the task remains a challenge. In another word, when designing the novel soft robots, there are few work taking the task constraints into account.

**Figure 1:**
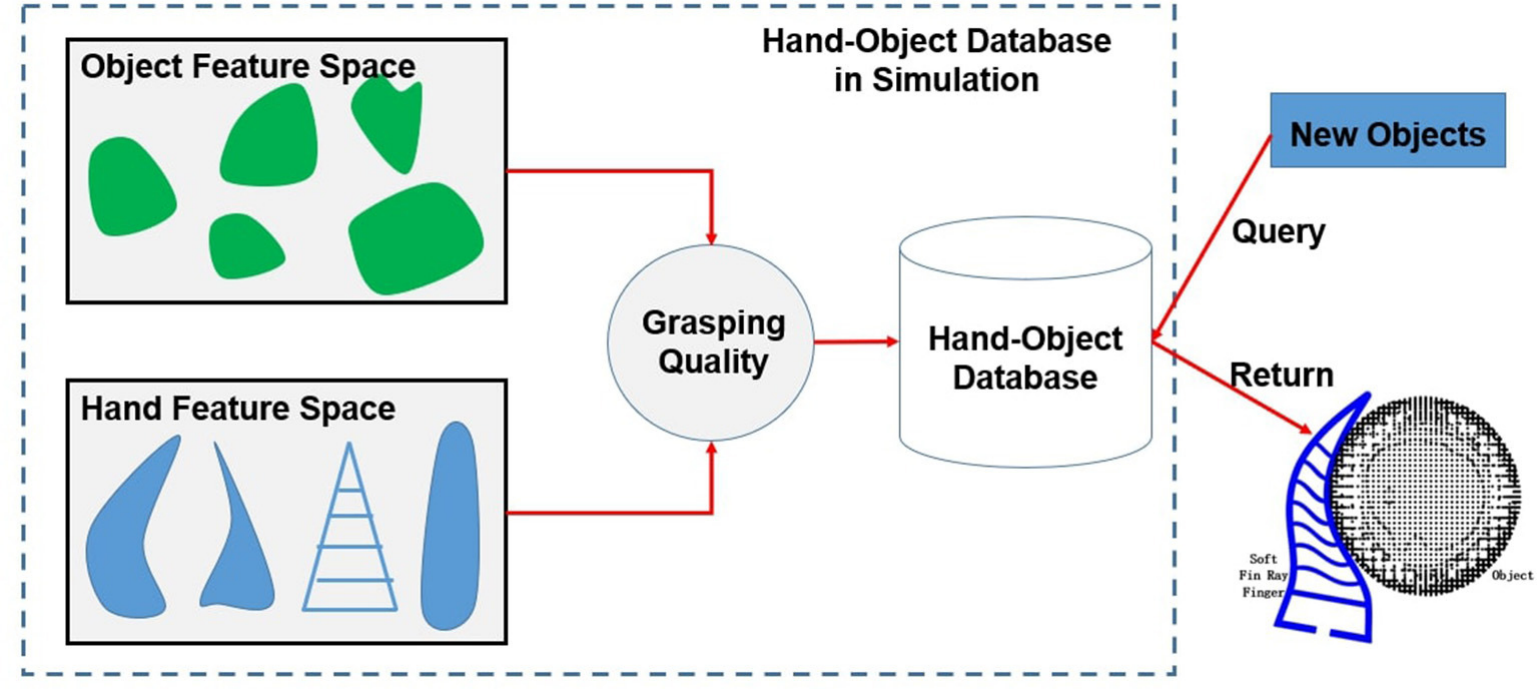
The pipeline of the proposed framework. The soft fin-ray hand and object feature are encoded and integrated with the grasp quality function in simulation. Given a new object, the best hand design parameters are obtained by querying the learned hand-object database.

In this paper, we address the problem of optimal geometry design for a fin-ray finger given an object to be grasped. The task constraint is encapsulated as a grasp quality function, which is a combination of the object shape and the geometry of the deformed finger. The object feature is modeled using superquadratics, which allows for a variety of inputs such as the point cloud and object CAD models. The grasp quality function is chosen as the winding number that represents how well the soft finger is surrounding the object surface. Given a new object, the best grasp quality with different hands can be quickly predicted using the object and hand features. An overview of the proposed framework is shown in Figure 1. The main contribution of this paper is two-fold: (1) We first propose a framework to learn the optimal fin-ray finger design for soft grasping. This can possibly open a new direction for the design of soft hands considering the task to be done, which is important to move soft robots from lab to real world. (2) For soft grasping, we propose to use the winding number to empirically estimate the quality of the obtained grasp. This quality function is not limited to fin-ray finger, but also can be used for many other types of soft robots.

The paper is organized as follows: section 2 provides a review of the related work, mainly focusing on the modeling of soft hands and the evaluation of soft grasping. Section 3 gives an approach to object and hand feature encoding, along with an empirical grasp quality function. Implementation details and experiments results are described in section 4, followed by a discussion of future directions and conclusion in section 5.

## 2. Related Work

Soft hand and soft grasping have been extensively studied during the past decades. The studies can be roughly divided into two sub-areas. The first one is trying to use new materials and structures to design various new hands and robots, which are different from previous robots that are built from rigid material including motors and links. Another direction is mainly focusing on the planning of compliant grasping with soft hands. The performance of the final grasping is usually demonstrated by the real-world grasping examples. There is no grasp quality function to guide the search for optimal grasps. The remaining part of this section will discuss the work on soft hand design and the performance of soft grasping, respectively.

### 2.1. Soft Hands Design and Modeling

Many researchers have attempted to use different materials, structures, sensors, and actuators to design novel robots and hands. Hannan and Walker (2003) developed a continuum style robot mimicking an elephant's trunk, where the kinematics is formulated using curvatures of the robot's shape. A universal gripper is developed based on jamming of granular material, with the goal to pick up unfamiliar object of widely varying shapes and surface properties (Brown et al., 2010; Amend et al., 2012). A wearable robotic glove is designed with soft actuators consisting of molded elastomeric chambers with fiber reinforcements that induce specific bending, twisting, and extending trajectories under fluid pressurization (Polygerinos et al., 2015a). The parameters are specifically selected to match the motion of human hands. Deimel and Brock (2016) describes the design and testing of an inexpensive, modular, under-actuated soft robot hand with pneumatically actuated fiber-reinforced elastomer fingers. The usage of various soft hands for exoskeletons is presented in Shahid et al. (2018). Internal sensing capability is further incorporated with a soft robot hand for robust proprioceptive grasping and object identification (Homberg et al., 2019). Abondance et al. (2020) presents a design of a prototype hand with dexterous soft fingers capable of moving object within the hand using several basic motion primitives.

There are some studies attempting to build the kinematic or kinetostatic models for soft robots and hands. The kinematics of constant curvature continuum robots is modeled and summarized in Webster and Jones (2010). A generic geometry-based framework is proposed to compute the deformation of soft robots within the range of linear material elasticity, namely linear stress–strain relation (Fang et al., 2020). The first-order dynamic modeling and control of soft robots is studied in George Thuruthel et al. (2020). For a fin-ray effect soft finger (Hosale and Kievid, 2010; Corporate, 2011; Pfaff et al., 2011), the kinetostatic model of a general multi-crossbeam finger is established (Shan and Birglen, 2020). A comprehensive survey on soft robotic gripper is given in Shintake et al. (2018) that categorize the soft gripping in three types: actuation, controlled stiffness, and controlled adhesion.

As discussed above, most of the previous work focus on the specific design of new soft hands and the analytical performance modeling of the designed hands. In this paper, one of the main motivations comes from the challenge that how to design a proper soft hand given the desired task requirement. To this end, two basic components are necessary: how to model the hand (in terms of kinematics, dynamics) and how to model the task requirements. In this paper, we choose to use the fin-ray finger as a testing example for our proposed framework, but also noticing that our approach is not limited to this specific type of fingers. Rather than using an analytic model to formulate the kinematics, the pseudo-kinematics of the finger is directly simulated using *Ansys Workbench*® due to its generality. For the task requirements, we choose stable grasping as the goal while some other more complex task requirements (e.g., object identification, dexterous manipulation) can be also considered. The performance of soft grasping is reviewed in the next part.

### 2.2. Quality of Soft Grasping

Unlike its counterpart of multi-fingered robotic hands where various metrics have been proposed to evaluate the quality of a given grasp, the grasping quality using a soft hand is usually much more difficult to evaluate. This is mainly due to the fact that the assumption of point contact is not valid in the case of soft grasping as mentioned in Shintake et al. (2018). The contact during soft grasping is continuously deformable in an infinity of possible shapes through interaction with objects. Several quality measures (Cloud Quality, Closure Index, and Net Force) are used in order to extract the grasp affordances for a soft hand (Bonilla et al., 2014). There are also some works that design the hand to simply mimic the structure or functionality of human hands (Deimel and Brock, 2016). Implicitly, in these cases the actual grasp quality is assumed to be the feasibility to achieve the human grasp taxonomy. Later, a co-design strategy is proposed to design the hand morphology with specific structure (Deimel et al., 2017). The grasp quality here is evaluated by a soft hand grasping simulator.

In this paper, we proposed to use the winding number to evaluate the quality of a soft grasp. Based on this quality function and the object shape geometry, the optimal finger design parameters can be selected. Compared with other grasping quality function, winding number inherently represents the total number of times that a curve travels around an object. This quality function is more generic and can be extend to many other soft robots with different kinematics.

## 3. Hand-Object Feature Encoding and Grasping Quality

As shown in Figure 1, the problem can be formulated as finding a mapping from a given task to a desired hand design, namely M:T→H(ϕ), where T and H represent the task requirement and hand design space, respectively. The task requirement in this paper is chosen as the quality of the final grasp and the hand design space depend on the design parameters of the hand ϕ.

### 3.1. Hand Feature Encoding

**Figure 2:**
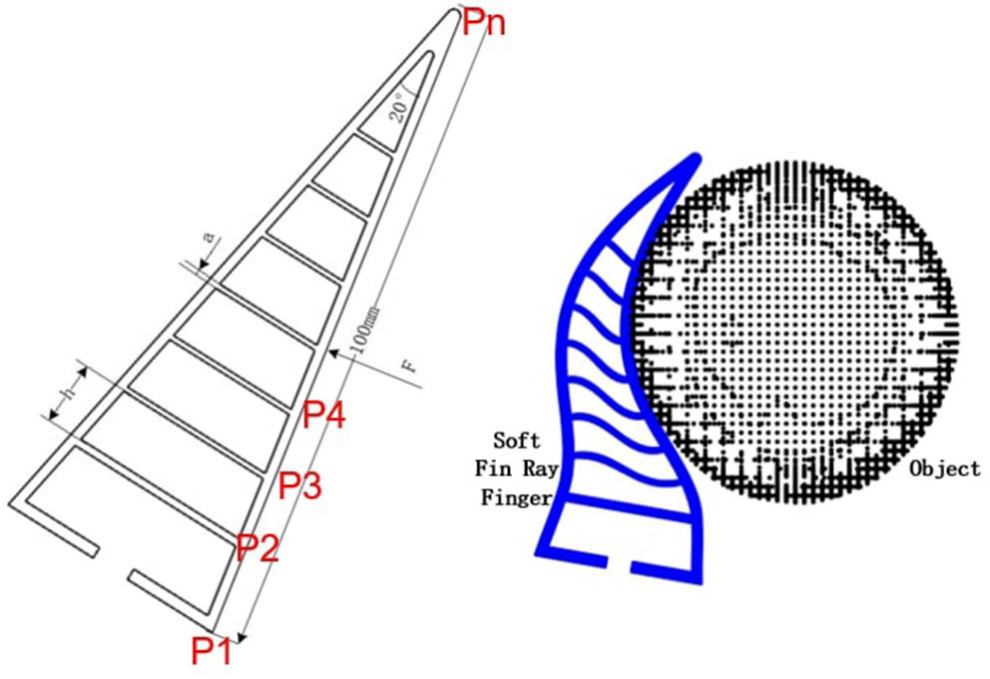
The fin-ray finger with its design parameters and its deformation during interaction with object.

A soft fin-ray finger is chosen as an example in this paper (see Figure 2), where the design parameters ϕ include the thickness *a* and the spacing distance *h*. Note that some other more complex parameters (angle, morphology) are not considered here for simplicity.

**Figure 3:**
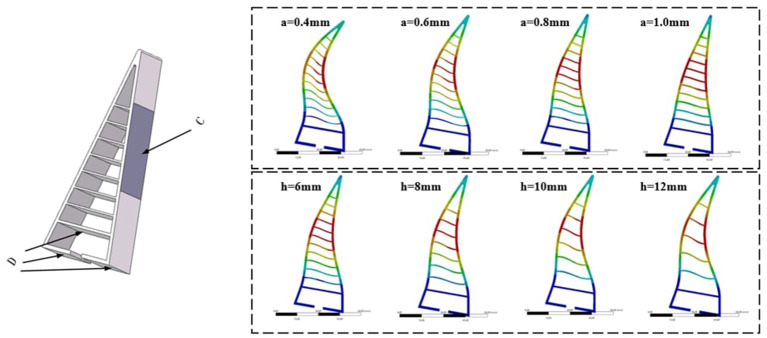
**(Left)** D represents the fixed constraints during simulation and C represents the area to apply forces. **(Right)** The resulting deformation with different design parameters.

During the hand object interaction, different selection of the design parameters will lead to different level of deformation and therefore different task performance. To compute these deformations, a dataset of the hand deformation is collected in simulation using *Ansys Workbench*®. The parameters *a*, *h*, and the applied force *F* are all chosen with fixed spacing and range. The material we used is TPU 95A with Young's modulus 26 MPa, yield strength 8.6 MPa, breaking strength 39 MPa, and Poisson ratio 0.48^1^. Some of the results are shown in Figure 2. After the simulations, a dataset of *N*_*h*_ hand feature is collected as: $D_{h}={\left\{F_{f}^{i},h^{i},a^{i},d^{i},x_{n}^{i},y_{n}^{i},z_{n}^{i}\right\}}^{i=1..N_{h}}$, where superscript represents the order of the hands. $F_{f}^{i}$ represents the contact force between the hand and the object, and *d*^*i*^ represents the maximal deformation. $x_{n}^{i},y_{n}^{i},z_{n}^{i}$ represents the coordinates of the nodes *Pn* after deformation as shown in Figure 3. As discussed above, note that some other analytical methods can be also used here to compute the deformation as presented in Fang et al. (2020).

### 3.2. Object Feature Encoding

A superquadratics model is used to represent the object shape (Jaklič et al., 2000), which is consisting of five parameters, namely *a*_1_, *a*_2_, *a*_3_, *e*_1_, *e*_2_. In our case, we only consider a two-fingered grasping and the section that *z* = 0 is considered as the grasping plane. Therefore, *e*_1_ and *a*_3_ are not required. Together with the allowed grasping force *F*_*o*_, the dataset of object feature can be encoded as: $D_{o}={\left\{F_{o}^{i},a_{1}^{i},a_{2}^{i},e_{1}^{i},e_{2}^{i}\right\}}^{i=1..N_{o}}$.

(1) $$\begin{array}{l} {\left({\left(\frac{x}{a_{1}}\right)}^{\frac{2}{e_{2}}}+{\left(\frac{y}{a_{2}}\right)}^{\frac{2}{e_{2}}}\right)}^{\frac{e_{1}}{e_{2}}}+{\left(\frac{z}{a_{3}}\right)}^{\frac{2}{e_{1}}}=1 \end{array}$$

Note that in this paper, we will assume that the parameters of the object feature are known in advance. However, it is still possible to extract these parameters from either object point cloud or the CAD model (El Khoury et al., 2012; El-Khoury et al., 2013; Li et al., 2016).

### 3.3. Soft Grasping Quality

**Figure 4:**
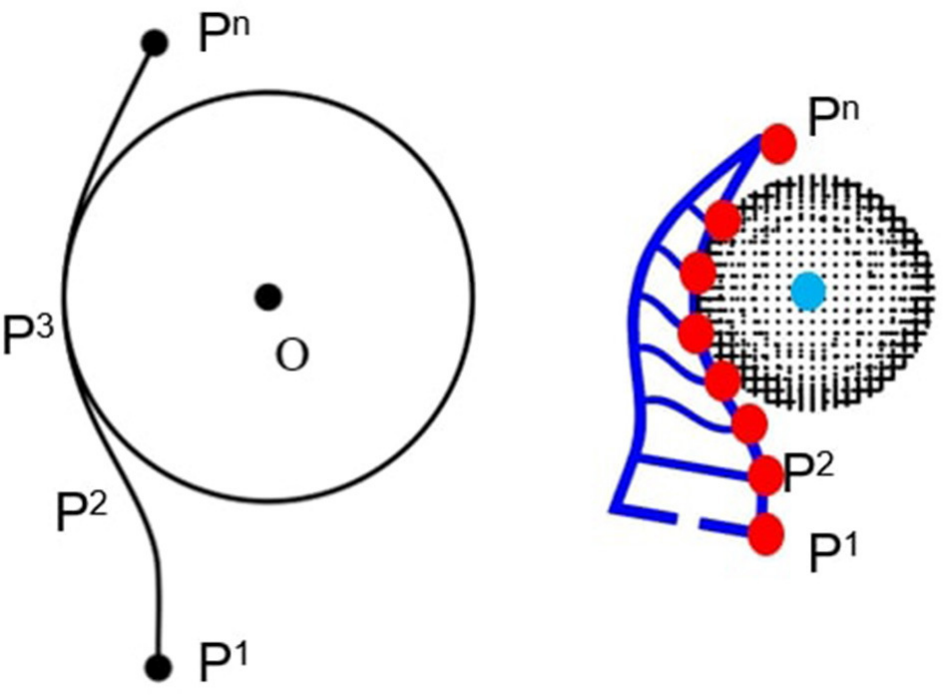
The illustration of winding number for soft grasping. The red dots represent the nodes of the beams, which are used for computing the winding number.

The task we consider here is stable soft grasping. To evaluate the quality of the grasp, a topological grasp quality measure is adopted, i.e., the winding number that counts the number of times that a curve wraps around a point (Pokorny et al., 2013; Li, 2016). In our case, this quality intuitively measures how well the fingers wrap around the approximated superquadratics. Mathematically, given a piecewise-linear curve that connects points, ${\text{p}}^{1},{\text{p}}^{2},{\text{p}}^{i}={\left[p_{1}^{i},p_{2}^{i}\right]}^{T}\cdots \;,{\text{p}}^{n}\in {\mathbb{R}}^{2}$, the winding number can be computed as (Hormann and Agathos, 2001), as illustrated in Figure 4,

(2) $$\begin{array}{l} w=\frac{1}{2\pi }\sum_{i=0}^{n-1}\{\tan^{-1}\left(\frac{{\text{p}}^{\text{i}+1}{}^{{}^{T}}({\text{p}}^{\text{i}+1}-{\text{p}}^{\text{i}})}{D_{i}}\right)\\ \;+\tan^{-1}\left(\frac{{\text{p}}^{\text{i}}{}^{{}^{T}}({\text{p}}^{\text{i}}-{\text{p}}^{\text{i}+1})}{D_{i}}\right)\} \end{array}$$

where $D_{i}=p_{1}^{i}p_{2}^{i+1}-p_{2}^{i}p_{1}^{i+1}$. Note that the winding number changes sign if the curve change direction. For our cases, we use two fin-ray finger for soft grasping. Therefore, we define the quality of a soft grasp G as,

(3) $$\begin{array}{l} w_{G}=|w_{l}|+|w_{r}| \end{array}$$

where *w*_*l*_, *w*_*r*_ corresponds to the winding number of the left and right finger, respectively. Since these two fingers are symmetric, $w_{G}=2|w_{l}|$.

### 3.4. Desired Parameters Selection

Given a dataset of the hand feature $D_{h}$ and the object feature $D_{o}$, the pairwise grasping quality can be computed using Equation (3). $w_{G}^{i,j},i=1..N_{h},j=1..N_{o}$ represents the grasping quality that grasping the *j*th object in $D_{o}$ using the *i*th hand in $D_{h}$. For *j*th object in the dataset, we can choose the best hand design as $\text{arg }{\text{min}}_{i}\;w^{i,j}$. The similarity of a new object and the object dataset is defined as:

(4) $$\begin{array}{l} Q^{new}=\min\left\{\alpha \|F_{o}^{i}-F_{o}^{new}\|+\beta \overset{2}{\underset{k=1}{\sum }}\left(\left\|a_{k}^{i}-a_{k}^{new}\|+\|e_{k}^{i}-e_{k}^{new}\right\|\right)\right\} \end{array}$$

where α and β are the scaling parameters. Given a manually selected threshold σ, if $Q^{new}<\sigma$, this new object is considered as similar enough to the objects in the dataset and the hand design is selected accordingly. When $Q^{new}>\sigma$, the object to be grasped is considered as unknown before, the procedure to find the optimal design parameters is summarized in Algorithm 1.

## 4. Experiments and Results

In the experiments, we demonstrated the results both in simulation and with real-world objects.

### 4.1. Results in Simulation

**Figure 5:**
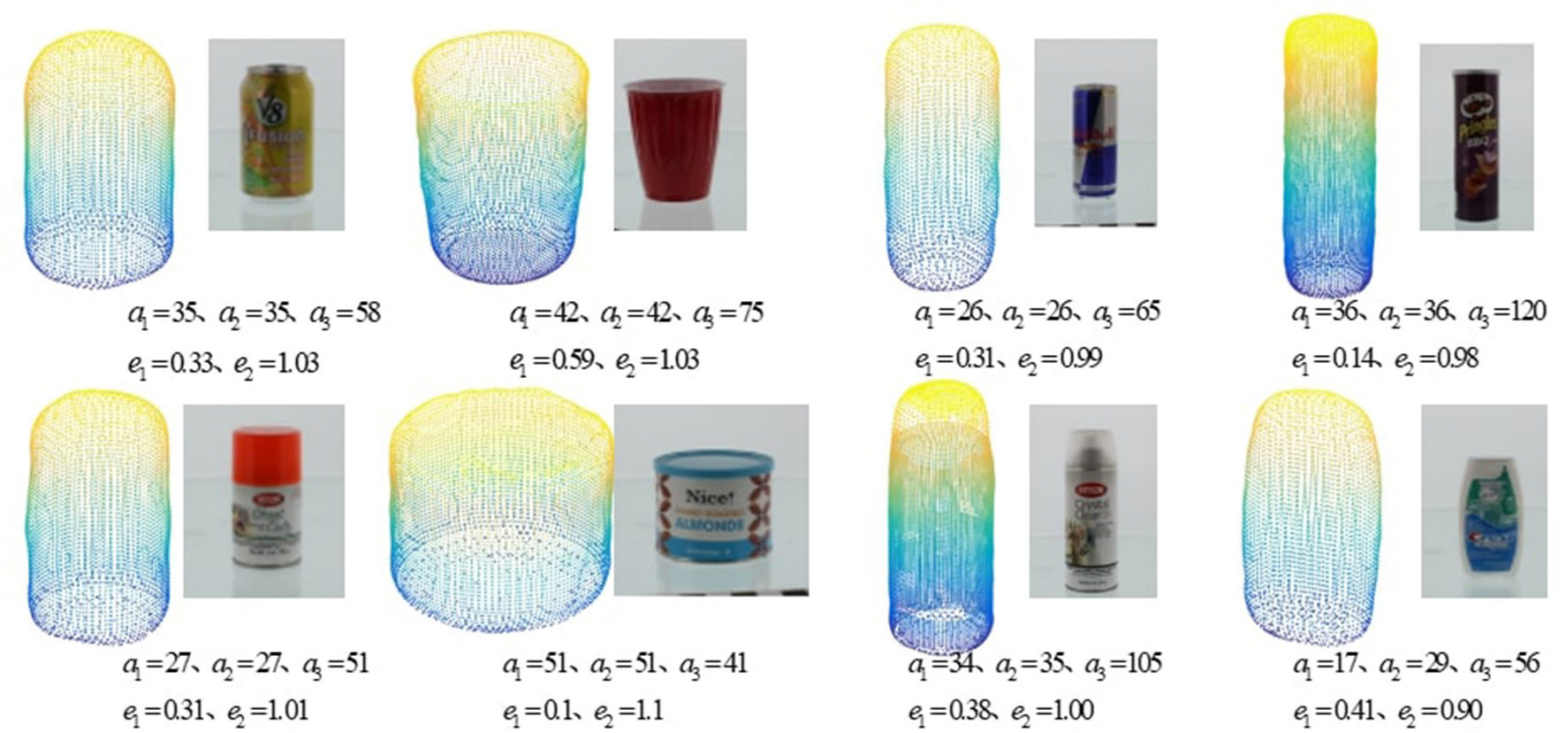
The YCB objects used in the experiments and their approximated superquadratics, including v8, red_cup,red_bull,pringles,krylon_short_cuts,honey_roasted_almonds,krylon_crystal_clear,crest_minty_fresh.

For the examples with simulated object, 8 objects from the YCB object dataset are used (Calli et al., 2015), as shown in Figure 5. The approximated superquadratics for each object is learned through constrained optimization (Jaklič et al., 2000).

**Figure 6:**
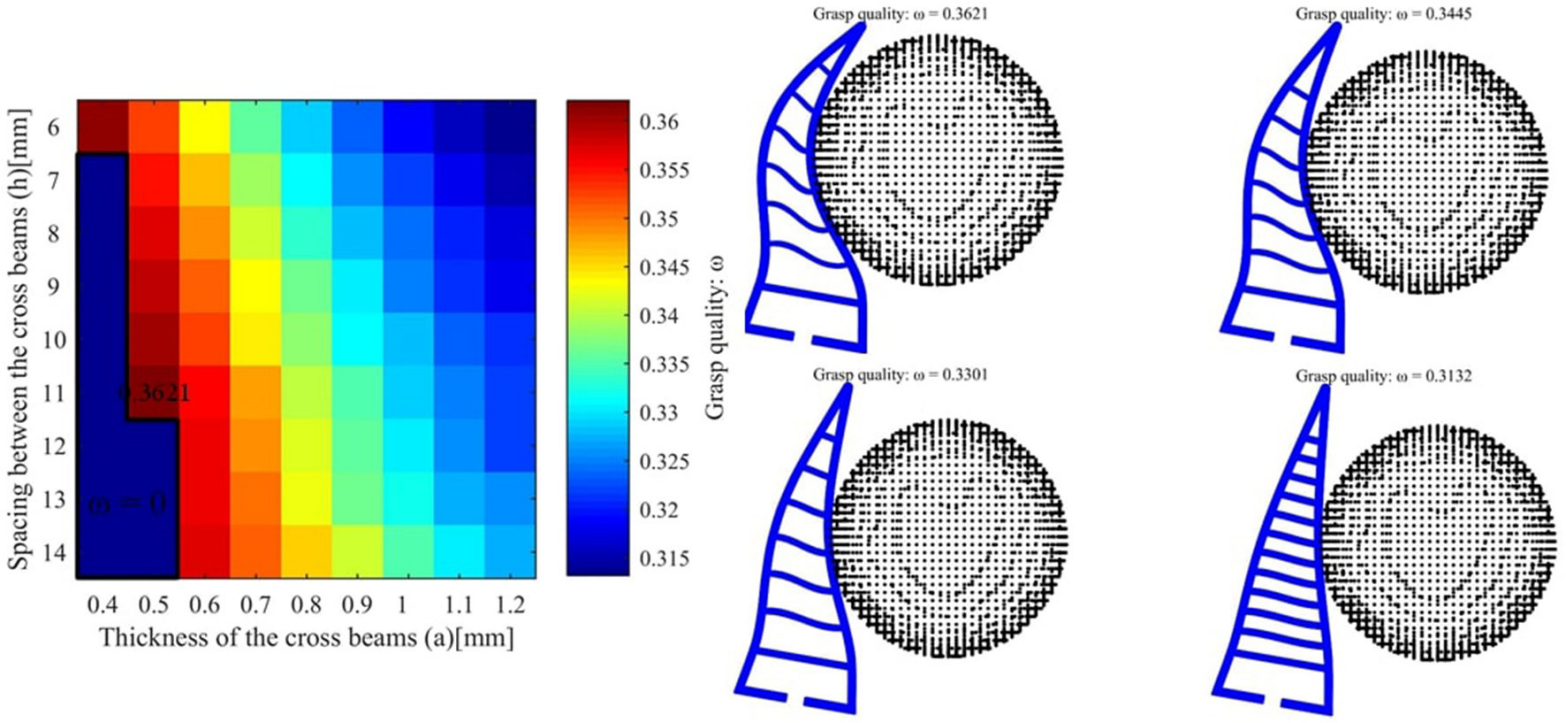
**(Left)** The grasping quality for object v8 with different hand design parameters. **(Right)** The hand object interaction for different final grasp qualities.

**Figure 7:**
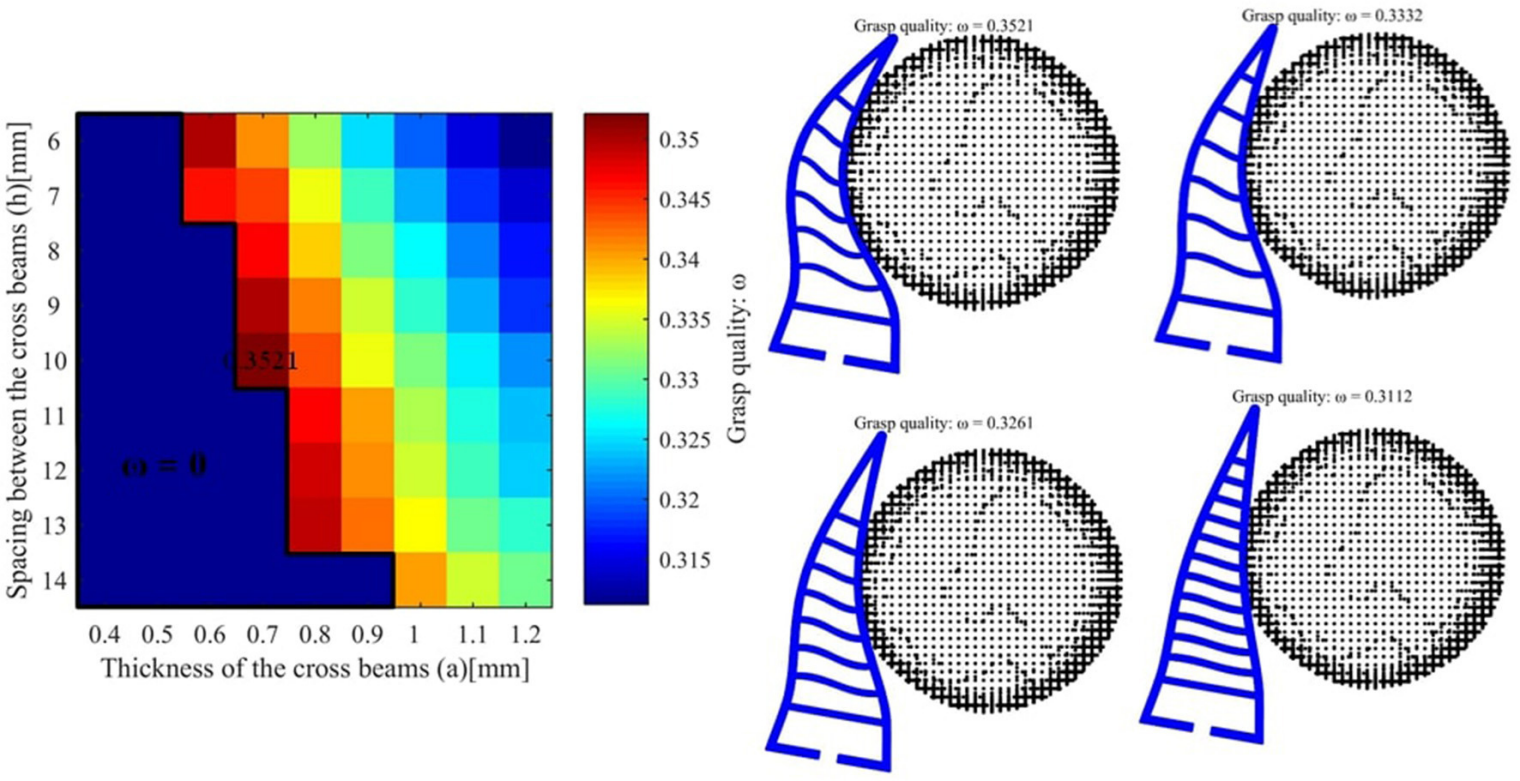
**(Left)** The grasping quality for object pringles with different hand design parameters. **(Right)** The hand object interaction for different final grasp qualities.

**Figure 8:**
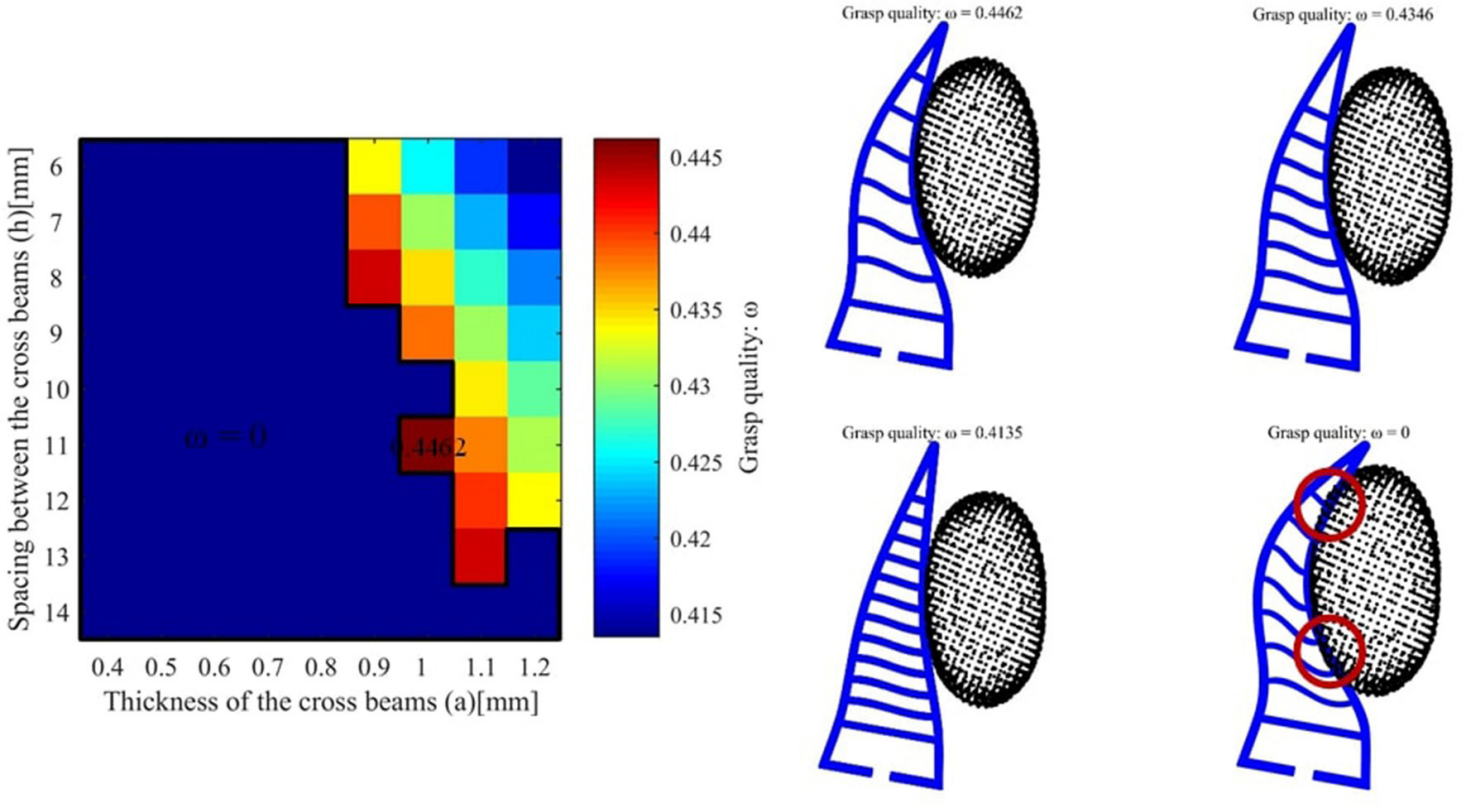
**(Left)** The grasping quality for object crest_minty_fresh with different hand design parameters. **(Right)** The hand object interaction for different final grasp qualities. The red circles stand for over-shot deformation and thus the quality is set to *w* = 0.

We take the first object v8 as an example and the desired force is set to 2*N*. The grasp qualities for different hand parameters is shown as follows (see Figure 6). The best grasp quality is 0.3621 and the corresponding parameters are *a* = 0.5*mm* and *h* = 11*mm*. For another object pringles, the desired force is set to 3*N* and the best hand design parameters (*a* = 0.7*mm* and *h* = 10*mm*) are shown in Figure 7. For another object cre_minty_fresh, the desired force is set to 3*N* and the best hand design parameters (*a* = 1.0*mm* and *h* = 11*mm*) are shown in Figure 8.

### 4.2. Results With Real-World Objects

**Figure 9:**
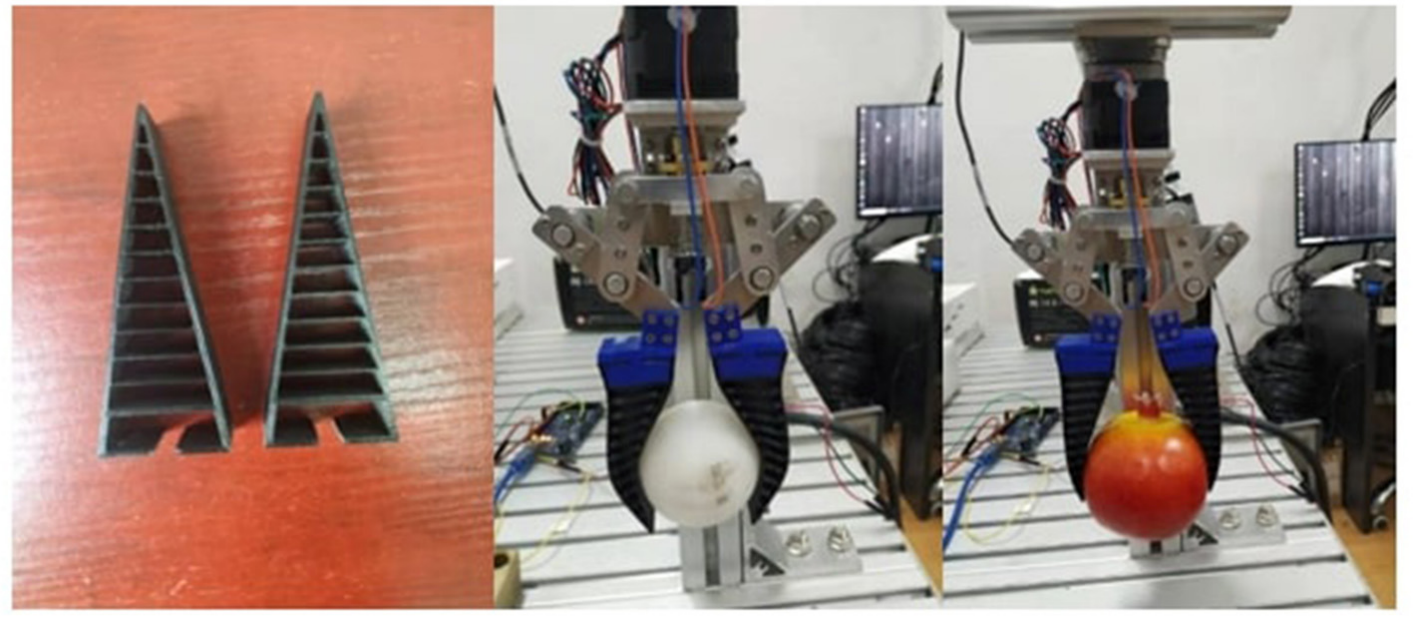
The 3D-printed fin-ray finger used for grasping two real-world objects.

The 3D-printed fin-ray finger used for grasping two real-world objects is shown in Figure 9. The true deformations for current examples are hard to measure. Therefore, we cannot directly compute the grasp quality in the real-world example. However, in the future work, we are planning to integrate tactile sensing with the soft finger, which allows us to estimate the grasp quality from previous experience (Li et al., 2014).

## 5. Discussion and Conclusion

### 5.1. Discussion and Limitations

The functionality of soft hands is highly dependent on the inherent compliance from the structure and the material. Therefore, it is important to select the proper design parameters for a given task or a set of tasks. The complete searching space of the design parameters is usually embedded in a high dimension space. This paper only studies the case of a fin-ray finger where the design parameters are simplified as thickness of the beam and the spacing between beams. The performance of the soft grasping is evaluated using winding number, which can be computed in simulation. A much larger grasping dataset can be created in the future to enlarge the search space for different tasks.

**Algorithm 1:**
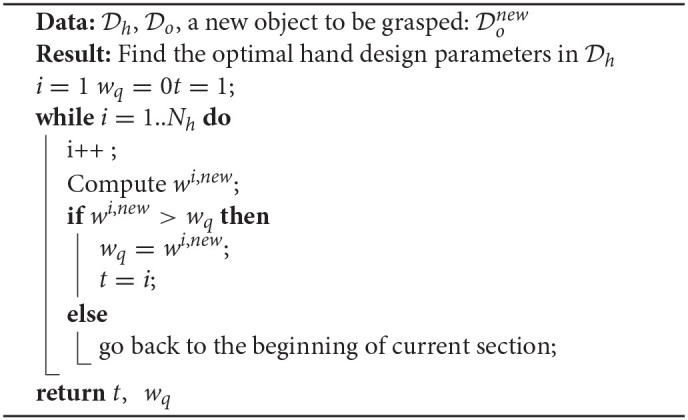
The algorithm to find the optimal hand design parameters for a new object.

There are some limitations of the current proposed framework. First, the task constraints for soft grasping is quantified using winding number. This is a geometric metric and it does not reflect the information from sensors and actuators. It is possible to co-design the structure and control algorithm together by taking into account the external sensing information (Deimel et al., 2017; Homberg et al., 2019). Second, the design space is simplified as a two-dimensional vector space in this paper. However, the morphology of the soft hand is a much more complicated space. It will be interesting to study the mapping between this morphology space to the final task performance. For example, in the fin-ray finger design, the beams are located with constant distance in parallel. It will be very useful to extend the space of the design parameters, especially by leveraging the power of big data and deep learning. This is part of our planned future work.

### 5.2. Conclusion

In this paper, we proposed a framework to learn the optimal fin-ray finger design for soft grasping. The hand feature is learned in simulation to encode the deformation of the finger during interaction. The object feature is represented using superquadratics and a topological metric is used to quantify the final task performance. Given a new object, the desired hand parameters can be quickly selected using the hand and object feature representation, under the guidance of the grasp quality function. For the future work, more complex hand design space and task quality function will be studied by leaveraging the power of big data and deep learning.

## Data Availability Statement

The original contributions presented in the study are included in the article/Supplementary Material, further inquiries can be directed to the corresponding author/s.

## Author Contributions

ZD designed the experiments and wrote the draft of this paper. ML served as adviser of this project and revised the draft accordingly. All authors listed have made a substantial, direct and intellectual contribution to the work, and approved it for publication.

## Conflict of Interest

ML was partly employed by the company Wuhan Cobot Technology. The remaining author declares that the research was conducted in the absence of any commercial or financial relationships that could be construed as a potential conflict of interest.

## Footnotes

^1^https://freddieflip.s3.us-west-2.amazonaws.com/uploads/sites/28/2018/03/28133640/Plural_Elasto95A_Data_Sheet-2.pdf
